# CAFs targeted ultrasound-responsive nanodroplets loaded V9302 and GLULsiRNA to inhibit melanoma growth via glutamine metabolic reprogramming and tumor microenvironment remodeling

**DOI:** 10.1186/s12951-023-01979-z

**Published:** 2023-07-08

**Authors:** Chen Ai, Xiao Sun, Shan Xiao, Lu Guo, Mengmeng Shang, Dandan Shi, Dong Meng, Yading Zhao, Xiaoxuan Wang, Jie Li

**Affiliations:** Department of Ultrasound, Qilu Hospital of Shandong University, Jinan, Shandong 250012 China

**Keywords:** Ultrasound, Tumor microenvironment (TME), Cancer-associated fibroblasts (CAFs), Glutamine metabolism, Metabolic reprogramming

## Abstract

Despite rapid advances in metabolic therapies over the past decade, their efficacy in melanoma has been modest, largely due to the interaction between cancer-associated fibroblasts (CAFs) and cancer cells to promote cancer growth. Altering the tumor microenvironment (TME) is challenging and elusive. CAFs is critical for glutamine deprivation survival in melanoma. In this research, we assembled a CAFs-targeted, controlled-release nanodroplets for the combined delivery of the amino acid transporter ASCT2 (SLC1A5) inhibitor V9302 and GLULsiRNA (siGLUL). The application of ultrasound-targeted microbubble disruption (UTMD) allows for rapid release of V9302 and siGLUL, jointly breaking the glutamine metabolism interaction between CAFs and cancer cells on one hand, on the other hand, blocking activated CAFs and reducing the expression of extracellular matrix (ECM) to facilitate drug penetration. In addition, ultrasound stimulation made siGLUL more accessible to tumor cells and CAFs, downregulating GLUL expression in both cell types. FH-V9302-siGLUL-NDs also serve as contrast-enhanced ultrasound imaging agents for tumor imaging. Our study developed and reported FH-NDs as nanocarriers for V9302 and siGLUL, demonstrating that FH-V9302-siGLUL-NDs have potential bright future applications for integrated diagnostic therapy.

**Graphical Abstract**

**Supplementary Information:**

The online version contains supplementary material available at 10.1186/s12951-023-01979-z.

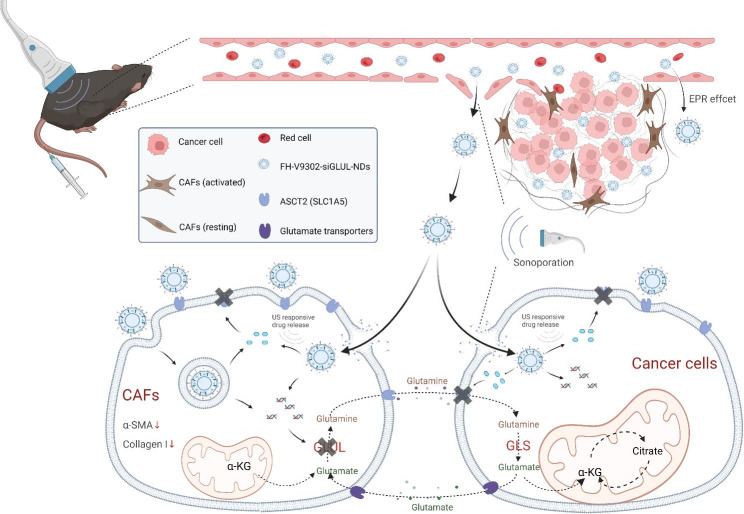

## Introduction

Tumor cells undergo metabolic reprogramming to meet the metabolic demands of energy and substances (amino acids, nucleic acids and lipids, etc.) for rapid proliferation [1], which is a unique metabolic feature of cancer [2]. Cancer cells are more dependent on glutamine than glucose [3]. Glutamine metabolism affects tumor cell proliferation, redox homeostasis, participation in macromolecular biosynthesis and the tricarboxylic acid (TCA) cycle [3], which is essential for anabolic growth and proliferation of cancer cells [4, 5], and also affects migration and invasion processes [4]. Cancer cells derive endogenous glutamine from glutamine synthetase (GLUL) and transport exogenous glutamine into the cell by SLC1A5 (also known as ASCT2) [6, 7]. Tumor proliferation stimulates upregulation of the glutamine transporter ASCT2/SLC1A5 and the GLUL [8]. V-9302 is an ASCT2 inhibitor which can block glutamine uptake [9, 10], and GLULsiRNA can restrain glutamine synthesis by suppressing GLUL. Both of them can inhibit tumor formation [11–14].

In the tumor microenvironment, the metabolism of multiple helper or stromal cells, such as fibroblasts, immune cells and endothelial cells, play crucial parts in tumor development and maintenance [15]. Cancer-associated fibroblasts (CAFs) are the major stromal cells in TME and are involved in all stages of cancer progression. CAFs can support nutrition to tumors by synthesizing or releasing amino acids [16, 17]. CAFs are major contributors to glutamine secretion [18]. High glutamine deficiency in cancer cells affects CAFs metabolism, increasing GLUL expression in CAFs, increasing glutamine synthesis to sustain the growth of these cancer cells [18], and harvesting glutamate from tumor cells to maintain CAFs growth [19]. CAFs compose and excrete abundant extracellular matrix (ECM), which is deposited in the TME and forms a physical barrier to drug treatment [20]. Because CAFs are involved in every stage of cancer development, targeting CAFs has become one of the key therapeutic platforms in the development of anti-cancer drugs.

**Fig. 1:**
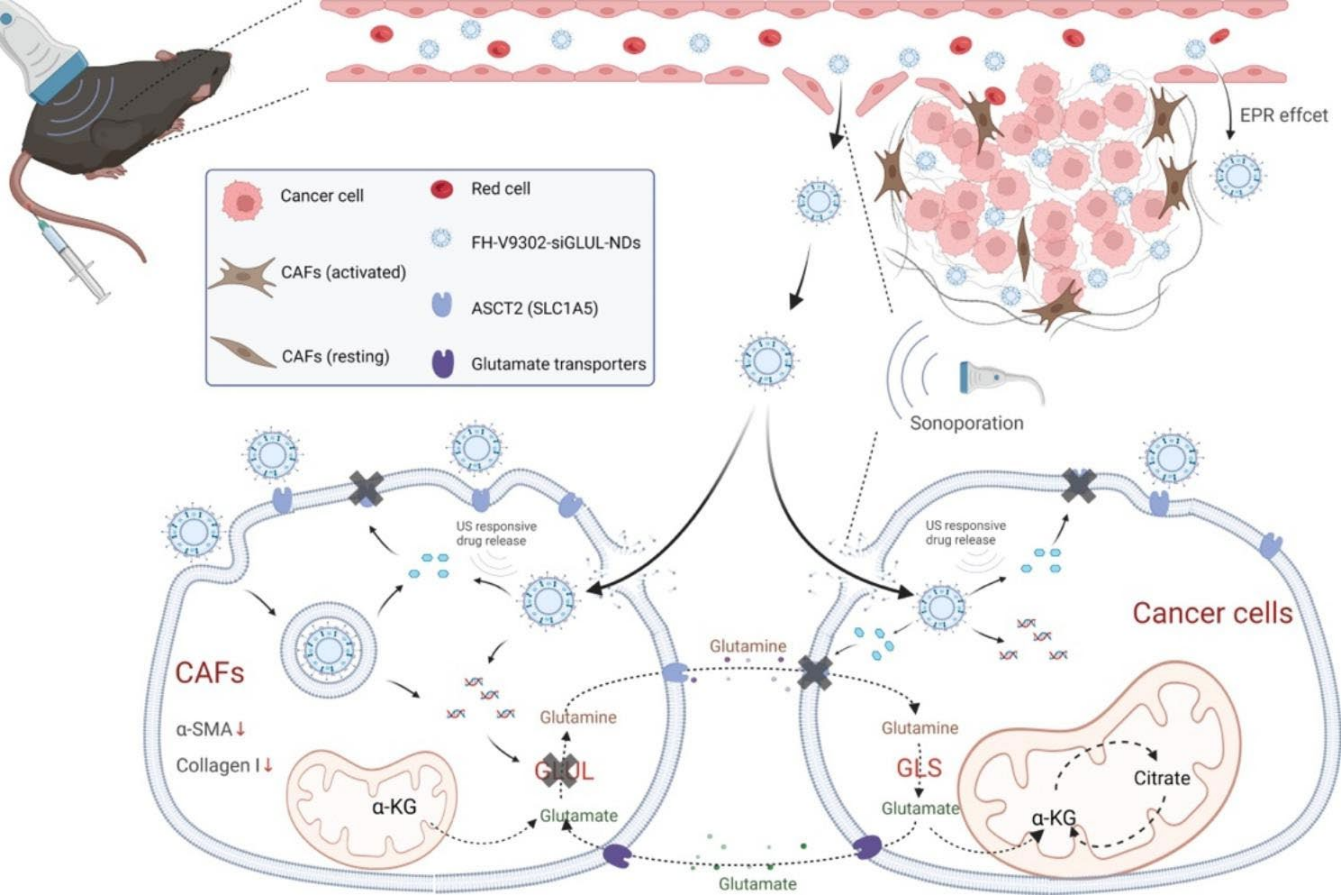
Schematic diagram illustrating antitumor effects by combining of UTMD and FH-V9302-siGLUL-NDs to cancer cells and CAFs

Tumor microenvironment is a complex metabolic system, therefore, drug design must take into account the non-cancerous cells in TME and the metabolic vulnerability of cancer cells [21]. Here, we successfully constructed controlled-release nanodroplets loaded with V9302 and GLULsiRNA, and enhanced drugs delivery using ultrasound-targeted microbubble disruption (UTMD). When nanodroplets bound to the cell surface, formed transient pores in the plasma membrane [22], allowing the payload to enter the cell membrane directly, achieving transient transfection of drugs and genes [23], and improving cell membrane permeability and enhancing cellular uptake [24] (Fig. 1). Due to the high binding affinity of FH peptide to tenascin C, the nanodroplets exhibit good CAFs targeting, targeting CAFs and releasing drugs in CAFs-rich TMEs. Our initial study was motivated by the possibility that ASCT2 inhibition and knockdown of GLUL might interrupt the metabolic coupling between B16F10 cells and CAFs. It has been found that dual inhibition of B16F10 cells and CAFs is important for matrix remodeling. Inhibition of activated CAFs could promote ECM degradation and improve nanodrug permeability. Targeting ASCT2 inhibition and GLUL knockdown in B16F10 cells and CAFs has the potential to reduce tumor growth and remodel the tumor microenvironment matrix. Indeed, the reduction of ECM in treated mice directly supports this mechanism.

## Materials and methods

### Reagents

Dipalmitoyl-sn-glycerol-3-phosphocholine (DPPC) and 1,2-distearoyl-sn-glycero-3-phosphoethanolamine-N-[amino (polyethylene glycol) − 2000] (DSPE-PEG2000- NH2) were obtained from Sigma-Aldrich (MO, USA). 3β-N, (N-(dimethylaminoethyl)-carbamoyl)- cholesteryl hydrochloride (DC-Chol) was purchased from Sigma-Aldrich (MO, USA). V9302 hydrochloride was purchased from MedChemExpress (USA). PFH was purchased from Aladdin (Shanghai, China). GLUL siRNA was purchased from Gene Pharma (China). Invitrogen Lipofectamine 2000 were obtained from Thermo Fisher Scientific (USA). Primary antibodies including rabbit anti-Collagen I, rabbit anti-α-SMA and rabbit anti-GLUL were obtained from Proteintech company (Wuhan, China). EdU-594 Cell Proliferation Kit and TUNEL Assay Kit were obtained from Beyotime (Haimen, China). Anti-Ki67 antibody was obtained from Servicebio (Wuhan, China).

### Cells

Mouse B16F10 malignant melanoma cells and NIH/3T3 embryonic fibroblasts were purchased from iCell Bioscience Inc (Shanghai, China). NIH/3T3 embryonic fibroblasts were activated into the CAFs model by treating with 5ng/mL TGF-β1 for 12 h.

### Animal tumor model establishment

Female C57 mice (SPF class), 6–8 weeks old, purchased from the Pengyue laboratory animal center (Jinan, China). All animal related studies were approved by the Research Ethics Committee of Qilu Hospital of Shandong University and carried out in accordance with the U.K. Animals (Scientific Procedures) Act (1986). The mouse was inoculated with mixed B16F10 cells (0.5 × 10^6^) and CAFs cells (1 × 10^6^) in a ratio of 1:2 into C57 mice.

### Preparation and characterization of nanodroplets

DPPC, DSPE-PEG2000-FH and DC-Chol were weighed in a molar ratio of 5:2:2, added to a tube with 0.2 mL of propylene glycol, and the propylene glycol was evaporated in a water bath at 60 °C. Then added 0.2 mL of hydration solution (glycerol: PBS = 1:9) to preheat to 37 °C and continue heating at 60 °C for about 15 min until the mixture was clear and transparent, then let it cool to room temperature. V9302 and 0.2 mL of PFH colostrum under ice bath conditions was added. Then, the mixture was emulsified by ultrasonic probe (VCX150, Sonics & Materials Inc, USA) in an ice bath at a power of 60 W for 5 min (10s working and 10s resting in turn). Nanodroplets (NDs) wrapped PFP were obtained and diluted with PBS after centrifugation at 12,000 r.p.m. for 10 min and discarding the supernatant. The FH-V9302-NDs was incubated with siGLUL for 30 min at 4 °C and centrifuged at 12,000 r.p.m. for 10 min to obtain FH-V9302-siGLUL-NDs in the lower layer and free siGLUL genes in the upper layer. The concentration of free genes in the upper suspension was measured at an absorption wavelength of 260 nm. Gene binding rate of nanobubbles = (total genes - free genes)/total genes × 100%. The particle size and ζ potential of nanodroplets were assessed using dynamic light scattering (DLS) (DelsaTM Nano C, Beckman Coulter, CA, USA). Then, the morphology of nanodroplets was characterized by transmission electron microscopy (TEM) (JEOL, Tokyo, Japan).

### Gel retardation assay

The siGLUL binding affinity of the nanodroplets was evaluated by gel electrophoresis at 120 V for 15 min and imaged by a Bioimage system (ChampChemi 620 Plus, Beijing, China). The optimal amount of formulation to form a complex with siRNA was determined by electrophoresis of non-binding siRNA on agarose gel electrophoresis. The optimal volume ratio of cationic lipid to siRNA was selected, and no bands of free siRNA were detected as optimal.

### In vitro drug release

The in vitro release of V9302 was evaluated by dispersing nanodroplets suspension in tubes. The tubes were shaken on a shaker (150 rpm, 37 °C). Then at fixed time intervals, the concentration of V9302 was measured at 220 nm with a UV-Vis spectrometer (Thermo Fisher, MA, USA). Accumulative release of V9302 (%) = amount of V9302 released / amount of V9302 initially encapsulated in FH-V9302-NDs × 100%. After ultrasound (US) irradiation (0.5 W/cm^2^, 60 s, 1.0 MHz), the ultrasound induced drug release was evaluated by FH-V9302-NDs solution.

### Efficiency of gene silencing in vitro

Firstly, in order to verify nanodroplets gene silencing effect, Lipo2000-siGLUL-treated cells were used as positive control and PBS-treated cells were used as negative control. B16F10 or CAFs (2 × 10^5^/well) were seeded in 6 wells overnight, then the medium was replaced with serum-free medium. PBS was chosen as control and siGLUL (100 nM siGLUL) was transfected in B16F10 and NIH/3T3 cells with LipofectamineTM 2000 according to the manufacturer’s instructions. FH-siGLUL-NDs were incubated with cells for 15 min, then ultrasound stimulation (0.5 W/cm^2^, 60 s, 1.0 MHz) was added. After 6 h of incubation, the medium was replaced with complete medium and incubation continued for 48 h. Next, the gene silencing efficiency of the nanodroplets was verified by studying the degree of down-regulation of GLUL proteins. Then, different nanodroplets were prepared in order to verify their gene silencing effect. B16F10 or CAFs (2 × 10^5^/well) were seeded in 6 wells overnight, respectively, and replaced with new media containing different elements. Preparations containing siGLUL were incubated with cells for 15 min, then with or without the application of ultrasound stimulation (0.5 W/cm^2^, 60 s, 1.0 MHz). The cells were incubated for 48 h. Then total proteins were extracted and the concentration was quantified. All primary antibodies were diluted at 1:1000. Secondary antibodies were diluted at 1:5000. Finally, protein bands were visualized. The Image J software was used to measure the contents of each proteins band.

### In vitro cytotoxicity

B16F10 or CAFs cells were seeded in 96-well plates overnight. Then, the medium was replaced with medium containing different elements. After 24 h, the medium was discarded and the viability of cells was evaluated by CCK-8 assay.

### In vitro antitumor effect

B16F10 cells were incubated with different treatments (PBS, FH-V9302-NDs, FH-V9302-siGLUL-NDs, Free V9302, V9302-siGLUL-NDs + US, FH-V9302-siGLUL-NDs + US (0.5 W/cm^2^, 60s, 1 MHz)), PBS served as control. Cell viability was determined by replacing medium with DMEM containing 10% CCK-8 solution in 96-well plates. A microplate reader was used to measure the absorbance of each well at 450 nm.

Cell proliferation ability with different treatment was evaluated by using the EdU-594 cell proliferation kit. Finally, the stained cells were observed using fluorescence microscope.

Transwell assays was used to analyze the migration and invasion abilities of malignant melanoma cells. B16F10 cells were seeded on 6-well plates and given different treatments. The same number of treated cells were collected and added to the upper chamber with or without matrigel, while the lower chamber was loaded with DMEM medium containing 15% FBS. After 24 h of incubation, the transwell chambers was fixed and stained with crystal violet. Finally, migrating or invading cells in each cell chamber were counted and photographed under a microscope.

### Serum stability assay

The serum stability of free siGLUL and FH-V9302-siGLUL-NDs was compared and determined in PBS with 50% serum (FBS, fetal bovine serum) concentration. Sodium dodecyl sulfate (SDS) terminated the ribonuclease activity in each sample and displaces the siGLUL adsorbed by the nanocarrier by competitive anion adsorption. When all supernatants were taken out, the completeness of siGLUL in each sample was observed by agarose gel electrophoresis.

### Hemolysis assay

In this study, the hemolysis of mice blood was tested by observing the free V9302, blank FH-NDs and FH-V9302-siGLUL-NDs at different concentrations. Hematocrit suspension solution was added to each sample, and the samples were placed on a shaker at 37℃ for 30 min. The samples were removed and centrifuged, and the samples were photographed for comparison. The OD of supernatant was measured using a microplate reader at 541 nm. The hemolytic rate was calculated as follows: hemolytic rate (%) = (OD_sample_-OD_negative_)/(OD_posive_-OD_negative_) ×100%.

### CAFs targeting ability of the FH-NDs

In vitro, CAFs cells and NIH/3T3 cells were incubated with Dio-labeled FH-NDs or NDs. After one hour, the cells were gently washed three times. Fluorescence microscopy and fluorescence-activated cell sorting assays were used to assess the targeting effect.

C57 mice bearing B16F10 (0.5 × 10^6^) and NIH/3T3 (1 × 10^6^) cell co-implanted tumors model was established. In vivo, the targeting of FH-NDs was verified by frozen sections and IVIS spectral image system (PerkinElmer, Waltham, MA, USA). Tumor-bearing mice were randomly grouped (n = 3) and injected intravenously with 150 µL of Dil-FH-NDs or Dil-NDs, and then mice were photographed using an in vivo imaging system 4 h after dosing. Tumors were made into frozen sections, which were then observed and photographed using a fluorescence microscope.

### Ultrasound imaging

Contrast enhanced ultrasound imaging (CEUI) ability of FH-V9302-siGLUL-NDs was evaluated using ultrasound scanner apparatus (LOGIQ E9, GE, USA). For in vitro US imaging, The FH-V9302-siGLUL-NDs solution was added to a finger inspection model of a latex glove and soaked in degassed water of 37 °C, followed by ultrasonic treatment. The major parameters are as follows: 9 L linear transducer; center frequency of 9.0 MHz; mechanical index (MI) of 0.8; dynamic range of 60 dB; focus of 3.0 cm; depth of 4.0 cm.

For in vivo US imaging, the tumor-bearing mice were injected 150 µL of FH-V9302-siGLUL-NDs intravenously while obtaining the US images of tumor. The major parameters are: 9 L linear transducer; center frequency of 9.0 MHz; MI of 0.5; dynamic range of 48 dB; focus of 1 cm; depth of 2.0 cm.

### CAFs morphology observation

CAFs cells were treated with different elements (PBS, FH-siGLUL-NDs, FH-V9302-NDs, FH-V9302-siGLUL-NDs, Free V9302, V9302-siGLUL-NDs + US, FH-V9302-siGLUL-NDs + US (0.5 W/cm^2^, 60 s, 1 MHz)). The cells were fixed and rinsed, followed by Actin-Tracker Green (Beyotime Biotechnology, China) staining and DAPI staining. Subsequently, CAFs morphology can be directly observed by fluorescence microscopy.

### In vivo biodistribution assessment

C57 mice bearing B16F10 (0.5 × 10^6^) and NIH/3T3 (1 × 10^6^) cell co-implanted tumors model was established. The co-implanted tumors model was used to do the IVIS imaging. To observe the biodistribution of FH-V9302-siGLUL-NDs, mice were injected with Dil-labeled nanodroplets (150µL per mouse). After injection at predetermined intervals, the mice were sacrificed to harvest tumors and major organs for ex vivo imaging using the IVIS spectrum image system (PerkinElmer, Waltham, MA, USA).

### In vivo antitumor study and biosafety

C57 mice bearing B16F10 (0.5 × 10^6^) and NIH/3T3 (1 × 10^6^) cell co-implanted tumors model was established. When tumors reach 50 mm^3^, tumor-bearing mice were randomly divided into 7 groups (n = 5) : G1, Control; G2, FH-siGLUL-NDs; G3, FH-V9302-NDs; G4, Free V9302; G5, FH-V9302-siGLUL-NDs; G6, V9302-siGLUL-NDs + US; and G7, FH-V9302-siGLUL-NDs + US. Corresponding formulations were injected via the tail vein at an equivalent dosage of 25 mg/kg V9302 per mouse. US irradiation (1.0 W/cm^2^, 60 s, 1 MHz) was performed 4 h post injection. The treatments were administered four times every 2 days. Tumor volume and weight were monitored every 2 days after treatment. Tumor volume = 0.5 × width^2^ × length. At the end of the experiment, resected tumors were collected, tumor weights were measured, and HE, IHC, and TUNEL assays were performed. Other vital organs were collected for H&E staining.

### In vivo TME remodeling

When tumors reach 50 mm^3^, tumor-bearing mice were randomly into 7 groups (n = 5). At the end of treatment, immunohistochemical staining of tumor was performed in order to verify the remodeling of the tumor microenvironment, including collagen I and α-SMA.

### Statistical analysis

All experiments were performed at least three times independently. Quantitative data were presented as mean ± SD. Statistical analysis was performed according to the student’s *t*-test or one-way ANOVA using SPSS software (version 26.0, USA). *p < 0.05 was considered to be statistically significant.

## Results and discussion

**Fig. 2:**
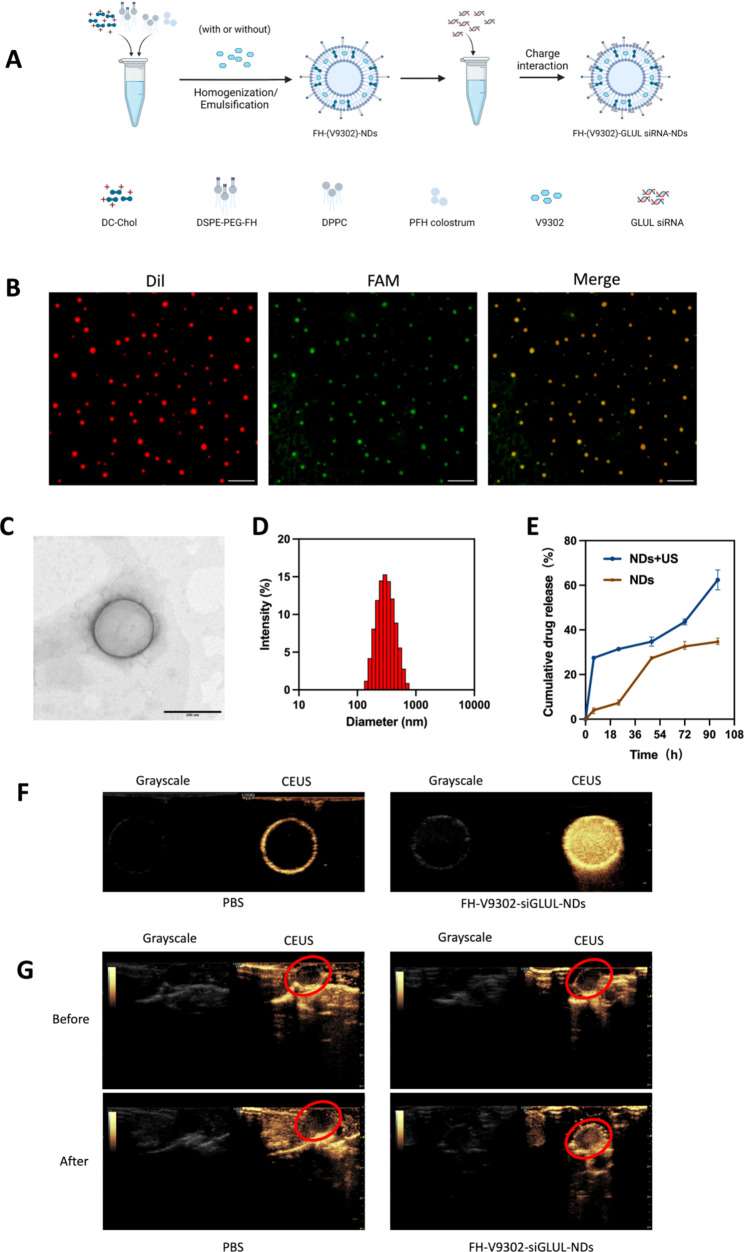
Preparation and characterization of the nanodroplets. **(A)** Preparation of the nanodroplets. **(B)** Fluorescence microscope diagram of FH-V9302-siGLUL-NDs. Scale bars: 10 μm. **(C)** Transmission electron microscopy (TEM) of FH-V9302-siGLUL-NDs at a volume ratio of 1:20 ratio (siGLUL: nanodroplets). Scale bars: 200 nm. **(D)** FH-V9302-siGLUL-NDs size distribution. **(E)** Drug release of FH-V9302-siGLUL-NDs with or without ultrasound stimulation (0.5 W/cm^2^, 60 s, 1.0 MHz). **(F)** US imaging in vitro. **(G)** US imaging in vivo. The red circle indicates the site of the tumor. All statistical data are expressed as means ± SD (n = 3)

### Characterization of nanodroplets

The DSPE-PEG-FH was assembled by the prior method [25, 26]. Based on previous studies [27], V9302 and siGLUL-loaded and FH peptide-modified sonicated nanodroplets, named FH-V9302-siGLUL-NDs (Fig. 2A). Fluorescence microscopy images of FH-V9302-siGLUL-NDs are shown in Fig. 2B. FH-V9302-siFAM-NDs-Dil was prepared by staining Dil and replacing siGLUL with FAM siRNA (siFAM). Red dots represented nanodroplets, green dots represented siFAM, and yellow dots represented the successful binding of FH-V9302-NDs-Dil and siFAM. The electron micrographs showed that the FH-V9302-siGLUL-NDs were regular spheres (Fig. 2C). The average diameter of FH-V9302-siGLUL-NDs was 301.1 nm, and average polymer dispersion index (PDI) was 0.132 (Fig. 2D). According to our study, the EE and LE of FH-V9302-siGLUL-NBs were 58.3% and 27.6%, respectively. The drug release of FH-V9302-siGLUL-NDs was also studied. Without exposure to US stimulation, the prepared FH-V9302-siGLUL-NDs exhibited slow drug release, only 34.7% of drug was released within 96 h. After exposure to US stimulation, V9302 was rapidly released, the cumulative release exceeded 62.4% in 96 h (Fig. 2E).

### The CEUI capability of nanodroplets

The CEUI capability of NDs can be visualized in real time. To evaluate the potential of FH-V9302-siGLUL-NDs + US as a CEUI agent, US imaging was performed in vivo and in vitro in grayscale and CEUS modes. In the in vitro experiments (Fig. 2F), there was no significant echogenic signal in the PBS group, in contrast, the echogenic signal of FH-V9302-siGLUL-NDs was significantly and substantially increased. In the in vivo experiments (Fig. 2G), the echogenic signal at the tumor site was almost unchanged after intravenous PBS injection. However, the intravenous FH-V9302-siGLUL-NDs group showed a strong increase in echogenic signal at the tumor site after injection. In conclusion, FH-V9302-siGLUL-NDs could improve contrast imaging and achieve an integrated mode of diagnosis and treatment.

**Fig. 3:**
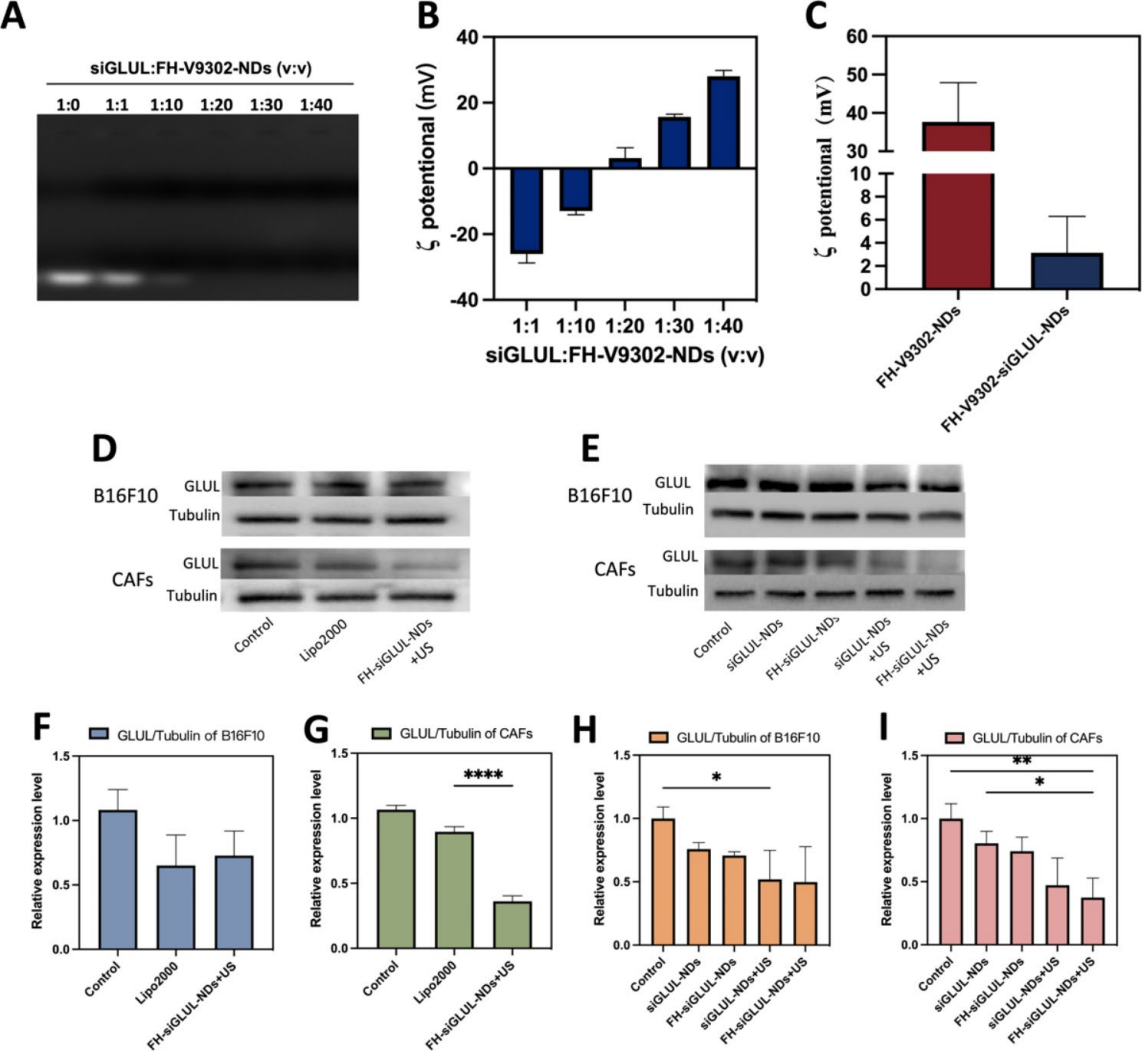
Characterization of FH-V9302-siGLUL-NDs and gene silencing efficiency. **(A)** Agarose gel electrophoresis retardation assay of FH-V9302-NDs with siGLUL in different volume ratios. **(B)** ζ potential changes of siGLUL with FH-V9302-NDs at different volume ratios (n = 3). **(C)** ζ potential of FH-V9302-NDs and FH-V9302-siGLUL-NDs (n = 3). **(D)** Western blot analysis of B16F10 and CAFs cells with different treatment knockdown GLUL levels. **(E)** Western blot analysis of different nanodroplets loading siGLUL knockdown GLUL levels. (F&G) Quantitative analysis of GLUL expression in B16F10**(F)** and CAFs**(G)** cells under different treatments. (H&I) Quantitative analysis of GLUL expression in B16F10 **(H)** and CAFs **(I)** cells treated with different nanodroplets loading siGLUL. *p < 0.05, **p < 0.01, ****p < 0.0001 (ANOVA test). All statistical data are expressed as means ± SD (n = 3)

### Characterization of FH-V9302-siGLUL-NDs and gene silencing efficiency

The siGLUL was diluted to 20 µM and positively charged nanodroplets were prepared with positive cholesterol, and siGLUL was prepared into nanodroplets at different volume ratios by charging interaction. FH-V9302-siGLUL-NDs were successfully prepared with different siGLUL: FH-V9302-NDs volume ratios (1:0, 1:1, 1:10, 1:20, 1:30 and 1:40), and Fig. 3A showed that FH-V9302-NDs could fully load siGLUL when the ratio was less than 1:20. The result showed the ζ potential of FH-V9302-siGLUL-NDs increases with the decrease of small disturbances (Fig. 3B). As the volume ratio increased from 1:10 to 1:20, the ζ potential changed from negative to positive, and siGLUL was fully loaded by positive FH-V9302-NDs at a ratio of 1:20. Therefore, a volume ratio of 1:20 ratio (siGLUL: nanodroplets) was the optimum ratio for the preparation of nanodroplets. The ζ potential of FH-V9302-siGLUL-NDs was + 3.15 ± 3.16 mV (Fig. 3C). The ζ potential of FH-V9302-NDs without siGLUL was + 37.67 ± 10.23 mV (Fig. 3C). Balancing the ζ-potential and siGLUL loading efficiency, a ratio of 1:20 was selected to synthesize the FH-V9302-siGLUL-NDs.

Above results suggested that the nanodroplets had the potential for gene silencing, which was further validated in subsequent experiments. The gene silencing efficiency of nanodroplets was investigated. CAFs and B16F10 cells transfected with siGLUL by LipofectamineTM 2000 (Lipo2000-siGLUL) for 6 h were used as positive control. Preparations containing siGLUL were incubated with cells for 15 min, then with or without the application of ultrasound stimulation (0.5 W/cm^2^, 60 s, 1.0 MHz). After 6 h of incubation, the medium was replaced with complete medium and incubation continued for 48 h. Western blot analysis was performed with differently treated B16F10 and CAFs cells (Fig. 3D). The results of their quantitative analysis showed that for the B16F10 cells group (Fig. 3F), Lipo2000 had a better silencing efficiency and the FH-siGLUL-NDs + US treatment was second only to Lipo2000. For the CAFs cells group, although Lipo2000 had a good gene silencing efficiency (Fig. 3G), the FH-siGLUL-NDs + US treatment had an even better gene silencing efficiency, reaching 64%. This could be related to the better cellular uptake of FH-siGLUL-NDs by CAFs. To further determine the efficiency of knockdown of GLUL, five groups of nanodroplets containing siGLUL were designed to investigate the effect of gene silencing on B16F10 and CAFs (Fig. 3E). The Western blot results were also quantified, and different treatment groups could lead to different decreases in GLUL levels on B16F10 (Fig. 3H) and CAFs (Fig. 3I). The targeting ability of FH-NDs and combined with ultrasound stimulation greatly inhibited the expression of GLUL in CAFs cells. Because CAFs cells had good cellular uptake efficiency for FH-NDs. Ultrasound stimulation greatly inhibited the expression of GLUL in B16F10 cells. Compared with the PBS group, the GLUL relative expression levels in the FH-siGLUL-NDs + US groups of B16F10 and CAFs were 0.50 ± 0.28 and 0.37 ± 0.15, respectively. However, in the absence of ultrasound stimulation, neither siGLUL-NDs nor FH-siGLUL-NDs significantly inhibited GLUL in B16F10 and CAFs cells. The above results suggested that ultrasound stimulation could significantly increase the silencing effect of GLUL in B16F10 and CAFs cells. The targeting ability could only improve the silencing effect of GLUL in CAFs cells, while it had no significant effect on the silencing effect of GLUL in B16F10 cells.

Western blot analysis was performed with differently treated B16F10 cells (Fig. S1A). Quantification of ASCT2 (Fig. S1B) by western blot showed that in the V9302-siGLUL-NDs + US and FH-V9302-siGLUL-NDs + US group, the relative expression of ASCT2 in B16F10 cells was significantly inhibited, with relative expressions of 0.39 ± 0.25 and 0.31 ± 0.10, respectively. The relative expression of ASCT2 in FH-V9302-NDs group with relative expressions of 0.89 ± 0.13. The above results can demonstrate that nanodroplets containing V9302 can inhibit the expression of ASCT2, while ultrasound stimulation can greatly inhibit the expression of ASCT2. The weaker inhibitory effect of the FH-V9302-NDs group on the expression of ASCT2 than the free V9302 group may be due to the slow release effect of the nanodroplets.

**Fig. 4:**
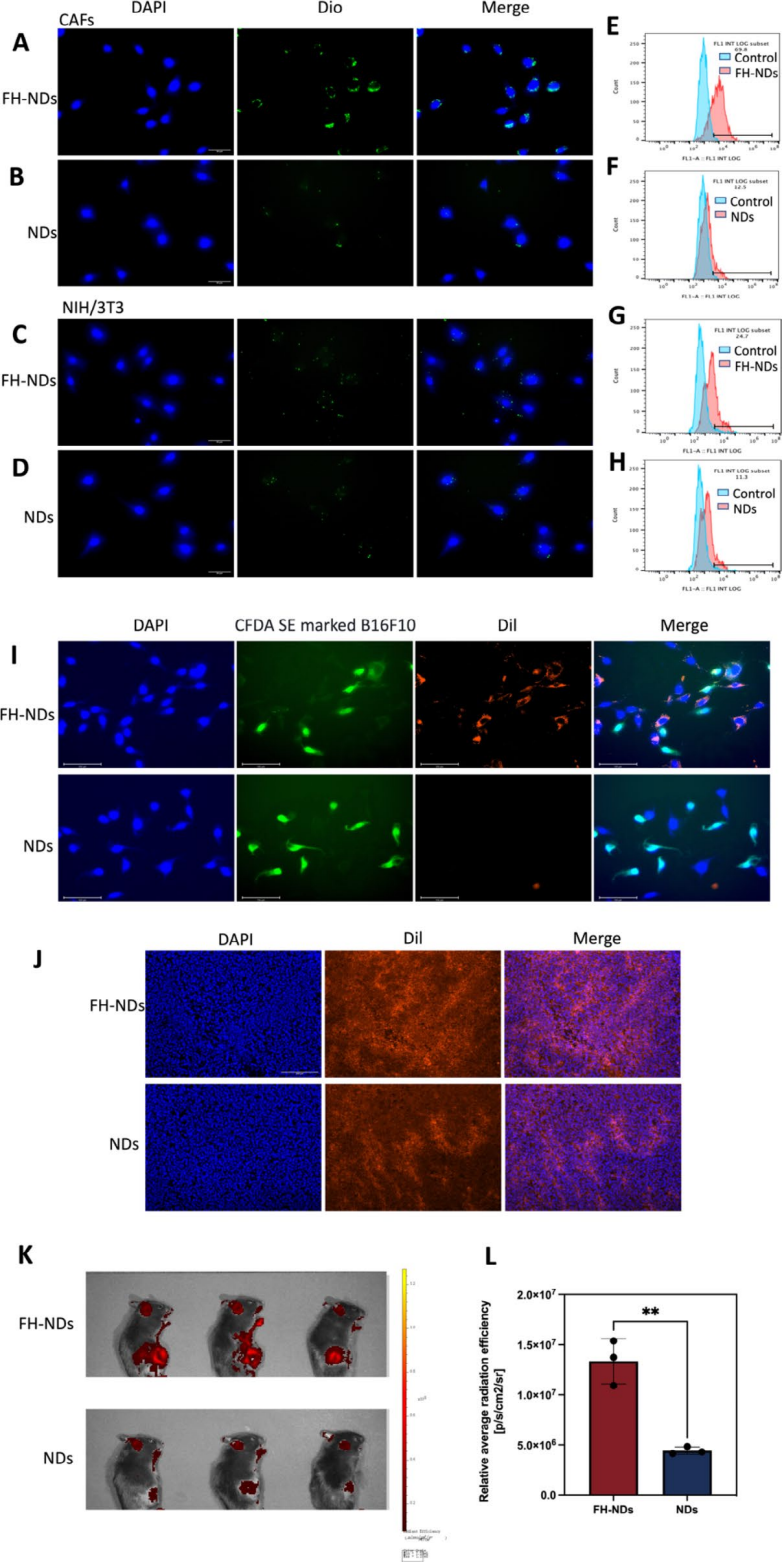
Targeted ability of FH-NDs. (A&C) Fluorescence microscope diagram of the targeted ability of FH-NDs. Scale bars: 30 μm. (B&D) Fluorescence microscope diagram of the targeted ability of NDs. Scale bars: 30 μm. (E-H) Flow cytometry measured the targeted ability of NDs and FH-NDs in CAFs and NIH/3T3 cells after incubation for one hour. **(I)** Fluorescence micrograph of the targeting ability of nanodroplets under co-culture conditions of CAFs with B16F10. Scale bars: 100 μm. **(J)** Targeting ability and accumulation of FH-NDs and NDs in tumor 4 h after injection of Dil-labelled nanodroplets. Scale bar: 200 μm. **(K)** Targeting ability of FH-NDs and NDs in mice 4 h after injection of Dil-labelled nanodroplets. **(L)** Quantitative analysis of targeting ability of FH-NDs and NDs in mice. **p < 0.01(*t*-test). All statistical data are expressed as means ± SD (n = 3)

### Targeted ability of FH-NDs

To verify the targeting ability of FH-NDs, Dio was chosen as a fluorescent probe. The targeting ability of FH-NDs was showed by fluorescence microscopy imaging and fluorescence-activated cell sorting. In fluorescence mode, nanodroplets labeled with Dio was green dot and nuclei stained with DAPI were blue. As illustrated in Fig. 4A and B, in CAFs groups, many FH-NDs aggregated on CAFs, while fewer NDs aggregated on CAFs. In the NIH/3T3 group, a small number of FH-NDs aggregated on NIH/3T3 and fewer NDs aggregated on NIH/3T3 (Fig. 4C and D). Fluorescence-activated cell sorting also showed, in CAFs groups, the targeted binding rate of FH-NDs was significantly higher than NDs, 69.37 ± 1.79% vs. 12.30 ± 0.44%, respectively (****p < 0.0001) (Fig. 4E and F). In contrast, for the NIH/3T3 groups, the binding rate of targeted FH-NDs was significantly higher than untargeted NDs, 21.20 ± 4.51% and 11.3 ± 0.86%, respectively (*p < 0.05) (Fig. 4G and H). We studied the cellular uptake of targeted and non-targeted nanodrops using the in vitro co-culture system. B16F10 was labelled with CFDA SE showing green fluorescence and then co-cultured with CAFs. The co-cultured cells were incubated with Dil-labelled FH-NDs and NDs for one hour respectively. The cellular uptake of the nanodroplets was observed by fluorescence microscopy photography (Fig. 4I). In the targeted FH-NDs group, nanodroplets were significantly aggregated in CAFs cells and rarely in CFDA SE labelled B16F10 cells. In the untargeted NDs group, there was no significant aggregation of nanodroplets in either CAFs or B16F10 cells. These results suggest that FH-NDs have a significant targeting effect on CAFs cells. The FH peptide has been shown to have an excellent high binding affinity for tenascin C, which is highly expressed in CAFs [28]. Furthermore, the targeting ability is correlated with tenascin C expression, which is low in NIH/3T3 cells and high in CAFs [29]. The result further suggested essential function of FH peptide in targeting binding to CAFs.

To further evaluate the in vivo targeting of FH-NDs, tumors transplanted to C57 mice were used as a model. It was observed by fluorescence microscopy that a significant intracellular red fluorescence was observed in frozen sections of tumors in the FH-NDs group, while less was observed in NDs group (Fig. 4J). As shown in Fig. 4K, the fluorescence signal of the FH-NDs group was more focused on the tumor than that of the NDs group, indicating the good targeting of FH-NDs. The fluorescence signal was quantified (Fig. 4L). In summary, FH-NDs have good active tumor targeting ability. The FH short peptide significantly increased the binding of targeted FH-NDs to tumors. The targeting ability of FH-NDs correlated with CAFs in tumors. Compared with NDs, FH-NDs could significantly accumulate in tumors in vivo, indicating a significant targeting ability, deep penetration and good retention effect. In our study, FH-NDs were actively targeted, which significantly enhanced the accumulation in tumor animal models.

**Fig. 5:**
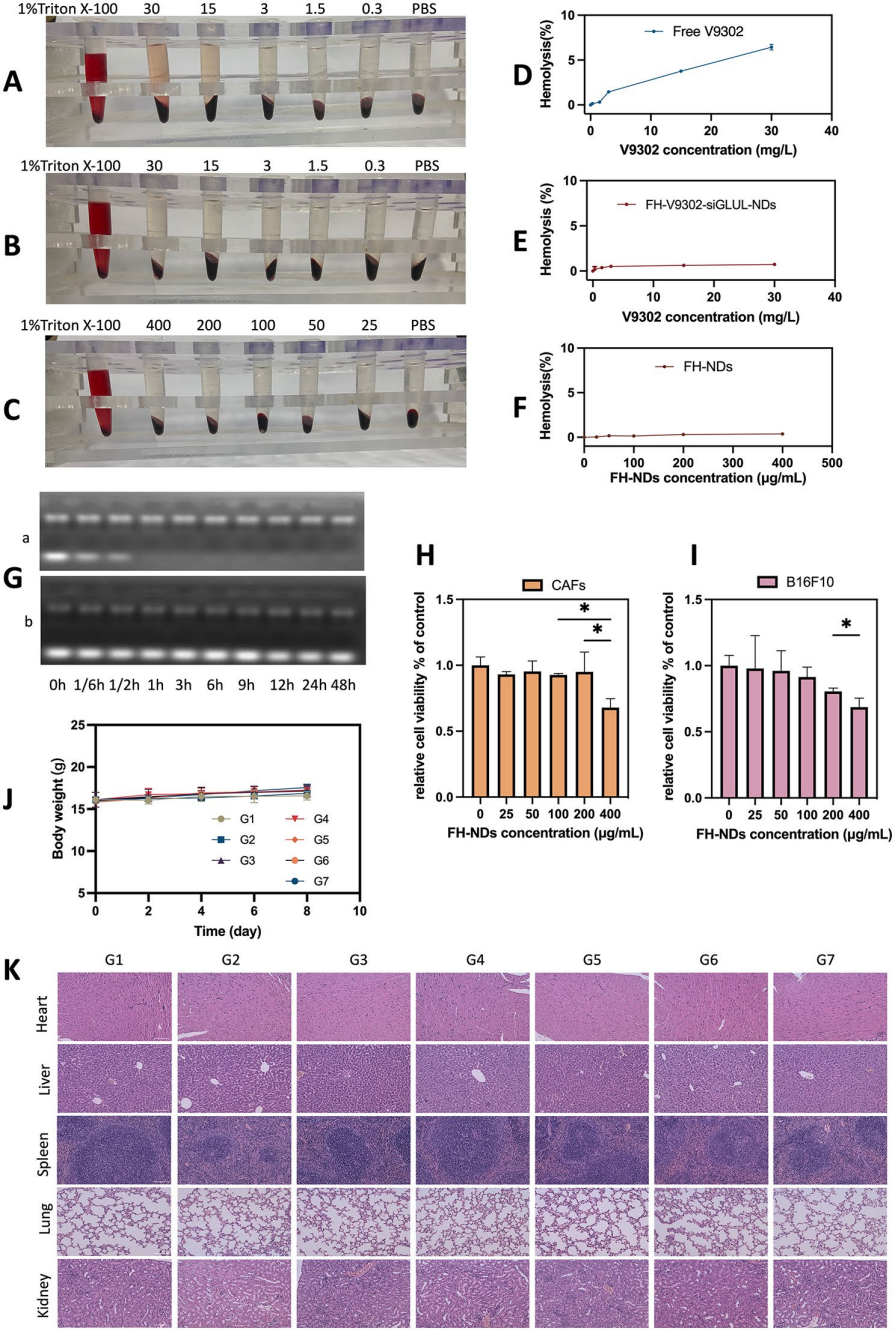
Safety evaluation. **(A)** Hemolytic behavior of Free V9302. **(B)** Hemolytic behavior of FH-V9302-siGLUL-NDs. **(C)** Hemolytic behavior of FH-NDs. **(D)** Statistics of hemolysis of Free V9302. **(E)** Statistics of hemolysis of FH-V9302-siGLUL-NDs. **(F)** Statistics of hemolysis of FH-NDs. **(G)** Agarose gel electrophoresis retardation assay of serum stability of naked siGLUL **(a)** and FH-V9302-siGLUL-NDs **(b)** in 50% fetal bovine serum. **(H)** Cytotoxicity of FH-NDs at different concentration in CAFs. **(I)** Cytotoxicity of FH-NDs at different concentration in B16F10. **(J)** Body weight changes of mice between treatments (n = 5). **(K)** H&E staining of major organs after intravenous injection of FH-V9302-siGLUL-NDs. Scale bar: 100 μm. *p < 0.05 (ANOVA test). All statistical data are expressed as means ± SD (n = 3)

### Safety evaluation

To confirm the biosafety of FH-NDs, the following experiments were performed. The biosafety of the nanodroplets was demonstrated by hemolysis tests. The results of blood erythrocyte lysis assays for free V9302, FH-V9302-siGLUL-NDs, and blank FH-NDs are shown in Fig. 5A, B and C, respectively. The results in Fig. 5D show that 30 mg/L of free V9302 produced an average hemolysis rate of 6.43% compared to 0.73% induced by the same concentration of V9302 in FH-V9302-siGLUL-NDs (Fig. 5E). In contrast, the blank FH-NDs had the lowest hemolysis rate which was 0.38% and had the best blood compatibility (Fig. 5F). Compared to FH-NDs, hemolysis rate increased after V9302 and siGLUL were encapsulated, but it was still much lower than free V9302. In addition, FH-V9302-siGLUL-NBs exhibited good serum stability. The free siGLUL band was significantly weakened within 10 min after incubation, and the band was completely absent by 1 h (Fig. 5G). This indicates that the free siGLUL was exposed to high concentration of FBS and was quickly and completely degraded by the enzyme. Observing the FH-V9302-siGLUL-NDs under the same FBS concentration incubation, siGLUL band intensity was somewhat weakened after incubation, bright siGLUL bands were still clearly observed until 48 h. The results indicated that positive FH-NDs had a protective effect on siGLUL. The cytotoxicity of blank FH-NDs was assessed by incubating CAFs (Fig. 5H) and B16F10 cells (Fig. 5I) with FH-NDs at different concentrations using the CCK8 assay. The results showed that significant cytotoxicity occurred when the concentration of blank FH-NDs reached 400µg/mL. The cell survival rates of the blank FH-NDs concentrations used in this study were all above 95%, indicating that FH-NDs used in our study were sufficiently safe.

To study the potential in vivo toxicity of nanodroplets, female C57 mouse models with tumors were treated differently. No significant changes in body weight of the mice were observed during the treatment period (Fig. 5J). After treatment, histological examination of vital organs showed no significant tissue damage (Fig. 5K). Overall, nanodroplets exhibited an acceptable biosafety profile.

**Fig. 6:**
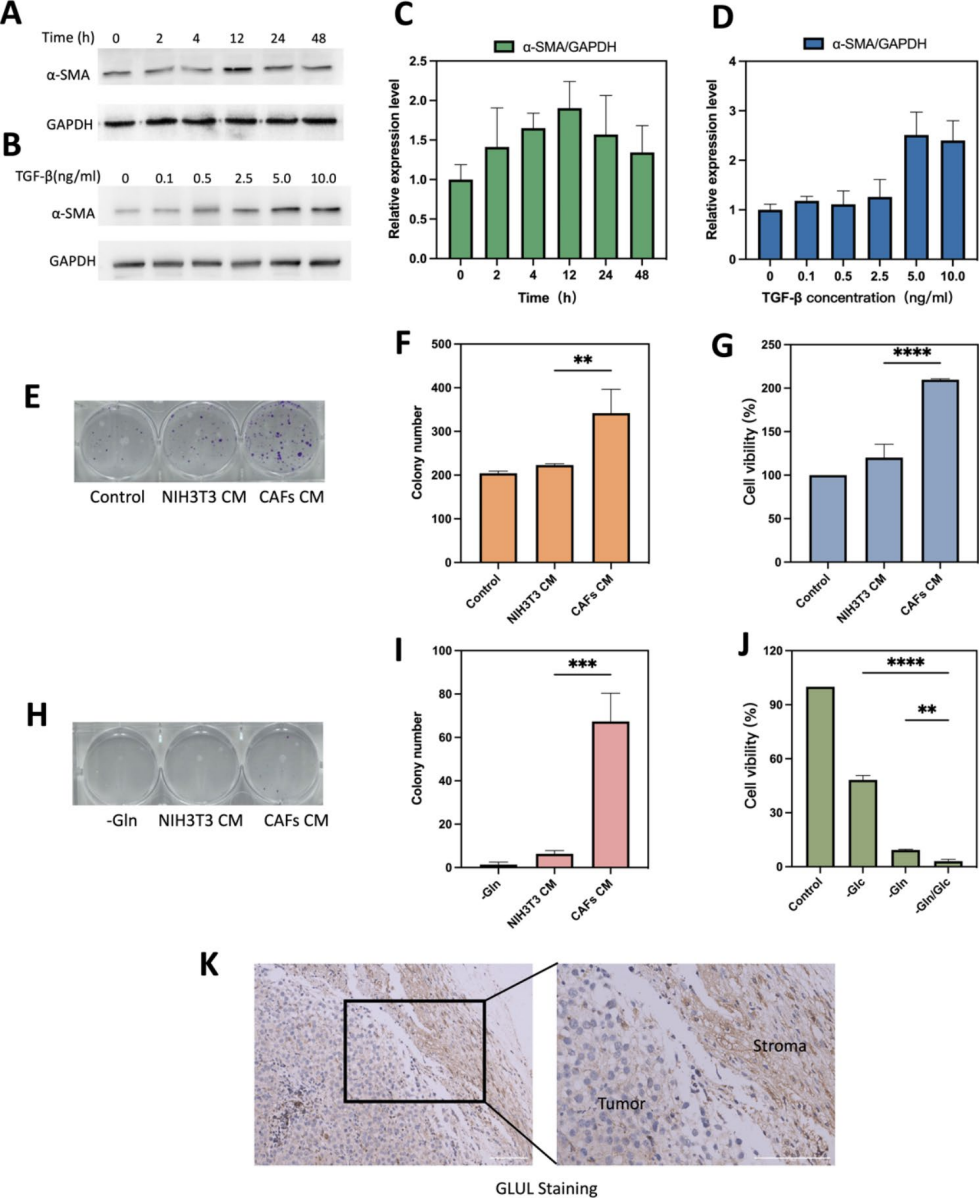
TGF-β1 induced CAFs and glutamine metabolism between CAFs and B16F10 cells. **(A)** Optimal time for induction of CAFs formation. **(B)** Optimal concentration of TGF-β for induction of CAFs formation. **(C)** Quantification of different treatment times of α-SMA/GAPDH. **(D)** Quantification of different treatment concentration of TGF-β of α-SMA/GAPDH. **(E)** Representative images and **(F)**histograms of the effect of normal medium, NIH/3T3 CM and CAFs CM on the colony forming ability of B16F10 after 10 days of incubation. **(G)** Cell viability of different CM on B16F10 cell growth after 24 h of incubation. (H&I) B16F10, NIH/3T3 and CAFs were cultured in Gln-deficient medium for 6 h. CAFs CM and NIH/3T3 CM were taken to replace the glutamate-free medium of B16F10 and the colony-forming ability was verified after 10 days. B16F10 cultured in glutamate-free medium was used as a control experiment. Representative images**(H)** and histograms**(I)** of the effect of different CM on colony-forming capacity of B16F10. **(J)**Cell viability of B16F10 cells with different condition medium. **(K)** IHC stained images comparing GLUL protein expression between stromal and tumor compartments. Scale bars: 100 μm. CAFs CM is conditioned medium derived from CAFs, NIH/3T3 CM is conditioned medium derived from NIH/3T3. -Gln is the glutamine deprivation condition, -Glc is the glucose deprivation condition, and -Gln/Glc is the simultaneous glutamine and glucose deprivation condition. **p < 0.01, ***p < 0.001, ****p < 0.0001 (ANOVA test). All statistical data are expressed as means ± SD (*n* = 3)

### NIH/3T3 successfully induced into activated CAFs cells and glutamine metabolism between CAFs and cancer cell

Mouse embryonic fibroblasts NIH/3T3 could be transformed into activated CAFs under certain conditions [30]. Markers for CAFs are usually smooth muscle actin (α-SMA), fibroblast activating protein (FAP), platelet-derived growth factor receptor (PDGFR) α/β, fibroblast growth factor receptor (FGFR) and tenascin C (TNC) [31]. As the most common used marker to label CAFs cells [32], α-SMA was chosen to verify the successful activation into CAFs in our study. As shown in Fig. 6A and B, expression level of α-SMA in TGF-β-stimulated serum-starved treated NIH/3T3 cells was significantly up-regulated at 12 h (Fig. 6C) or 5 ng/mL (Fig. 6D). Therefore, serum starvation treatment of NIH/3T3 cells at 5 ng/mL of TGF-β1 for 12 h was used as the experimental condition for mimicking the tumor microenvironment.

The results of plate clone formation experiments showed that the number of clones formed in the CAFs CM treated group was significantly higher than that in the NIH/3T3 CM treated group (Fig. 6E). CAFs CM could accelerate the proliferation of B16F10 and enhanced their proliferation ability. The difference was statistically significant (P < 0.01) (Fig. 6F). As shown in Fig. 6G, CCK8 assay provided similar results to cloning experiments, cell viability in the CAFs CM group was stronger than in the NIH/3T3 CM group (209.8 ± 0.95% vs. 120.1 ± 15.38%; *p* < 0.0001). All the above results demonstrated that serum-starved treated NIH/3T3 cells could be converted to functional CAFs under TGF-β1-stimulated conditions. The results of nutritional deprivation experiments showed that the cell survival rate of B16F10 was lower under glutamine deprivation conditions than under glucose deprivation conditions, 9.34 ± 0.34% and 48.24 ± 2.39%, respectively (Fig. 6J). The results and previous research all suggested that B16F10 relies more on glutamine metabolism than glycolytic metabolism [33]. And the results of colony genesis experiments confirmed that CAFs CM rescued cancer cells under glutamine-deficient conditions, while NIH/3T3 CM barely rescued, 67.33 ± 13.0 vs. 6.33 ± 1.54, respectively (Fig. 6H and I). May be due to that CAFs could upregulate its glutamine synthesis and secrete glutamine into TME [30, 34]. Tumor metabolic reprogramming results in altered glutamine metabolism between tumor and CAFs [35]. GLUL staining in mouse melanoma solid tumor tissues confirmed that GLUL expression was higher in fibrous stromal components compared to tumor cells (Fig. 6K). The higher expression of GLUL in CAFs also implied that CAFs could play a role in synthesizing glutamine (Gln) to supply tumor cells and maintain the growth of cancer cells under nutrient-deficient TME [13].

**Fig. 7:**
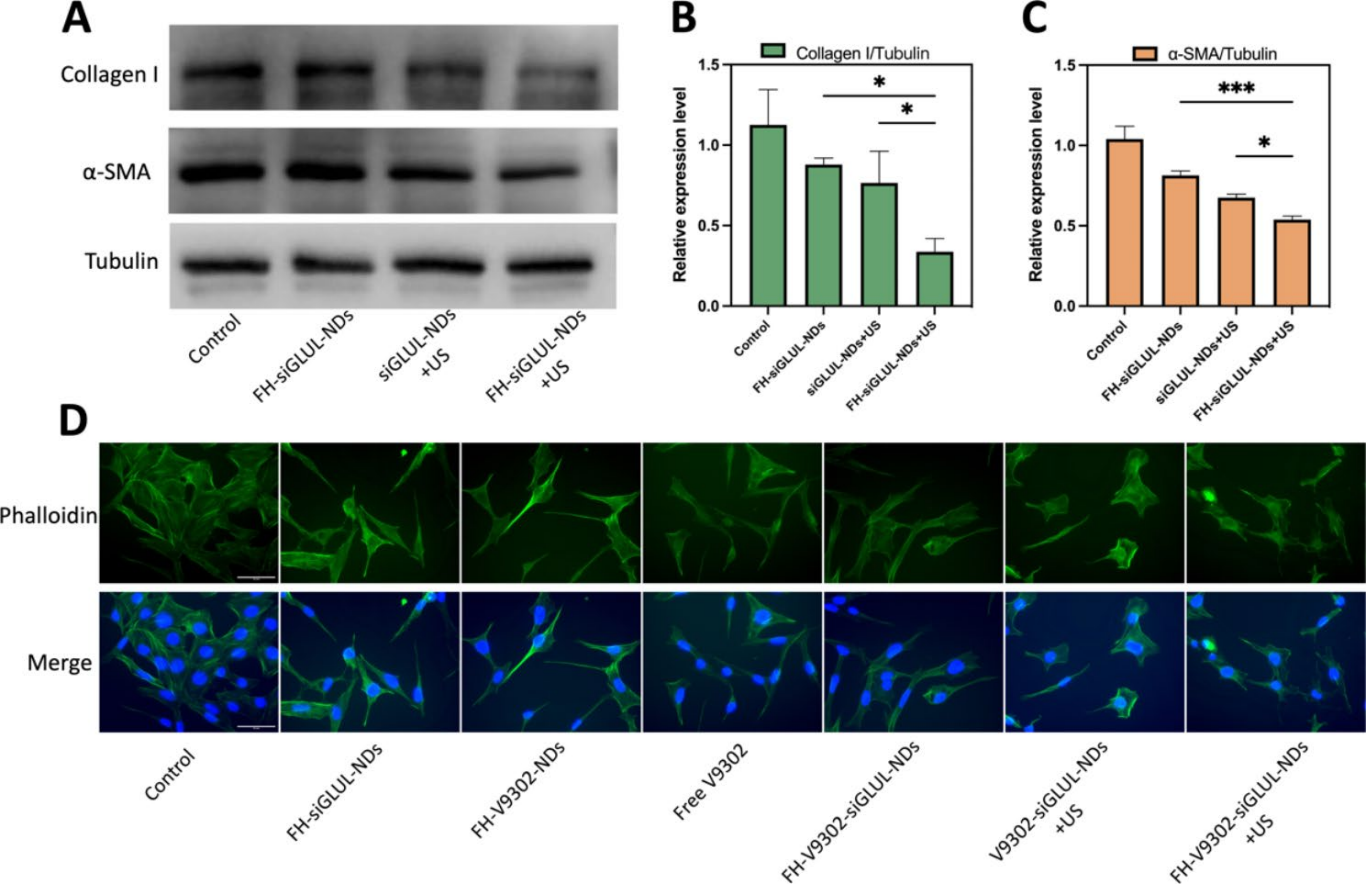
Inhibition of ECM and CAFs morphology after various treatments **(A)** Western blot analysis of collagen I and α-SMA after 48 h of CAFs treated with different elements containing siGLUL. **(B)** Quantitative analysis of collagen I expression in CAFs. **(C)** Quantitative analysis of α-SMA expression in CAFs. **(D)** Fluorescence microscope diagram of CAFs morphology 24 h after various treatments. Scale bar: 50 μm. *p < 0.05, ***p < 0.001 (ANOVA test). All statistical data are expressed as means ± SD (*n* = 3)

### Inhibition of ECM and CAFs morphology observation

It has been shown that blocking glutamine metabolism may severely disrupt the entire tumor metabolism, with a major impact on TME [28]. CAFs could increase the secretion of ECM, such as collagen I. And a large and dense ECM could act as a physical shield for the tumor, preventing drug penetration or infiltration into the tumor, leading to poor efficacy [20]. In this study, we found that the cytotoxicity of the nanodroplets containing V9302 on CAFs was less than that of B16F10 cells, and the IC50 values of free V9302 on B16F10 and CAFs cells were 33.52 µM and 69.26 µM, respectively. This indicated that V9302 did not kill CAFs directly, but change it from an active to a quiescent state, thus reducing the secretion of ECM [36]. Preparations containing siGLUL were incubated with cells for 15 min, then with or without the application of ultrasound stimulation (0.5 W/cm^2^, 60 s, 1.0 MHz). The cells were incubated for 48 h. The inhibitory effects of siGLUL-containing nanodroplets on α-SMA and collagen I were verified by western blot (Fig. 7A). Quantification of collagen I (Fig. 7B) and α-SMA (Fig. 7C) by western blot showed that in the FH-siGLUL-NDs + US group, the relative expression of collagen I and α-SMA in CAFs cells was significantly inhibited, with relative expressions of 0.34 and 0.54, respectively. The above results showed that siGLUL-containing nanodroplets could inhibit the formation of ECM, while ultrasound stimulation could promote the entry of siGLUL into cells and thus could significantly inhibit the formation of ECM. The ability of the targeted group to inhibit ECM formation was higher than that of the non-targeted group, indicating that the targeted combined with ultrasound treatment group had a stronger ability to eliminate ECM.

According to fluorescence microscopy imaging (Fig. 7D), simultaneous combined glutamine metabolic blockade and ultrasound stimulation resulted in significant changes in morphology of CAFs cells. The group containing V9302 changed CAFs from active to quiescent state, and CAFs morphology become long and lean. Combined siGLUL and ultrasound stimulation changed the cell morphology irregular and chaotic. These results demonstrated that ultrasound-responsive release of V9302 and siGLUL could regulate cell dynamics by reorganizing the morphology and could inhibit ECM deposition.

**Fig. 8:**
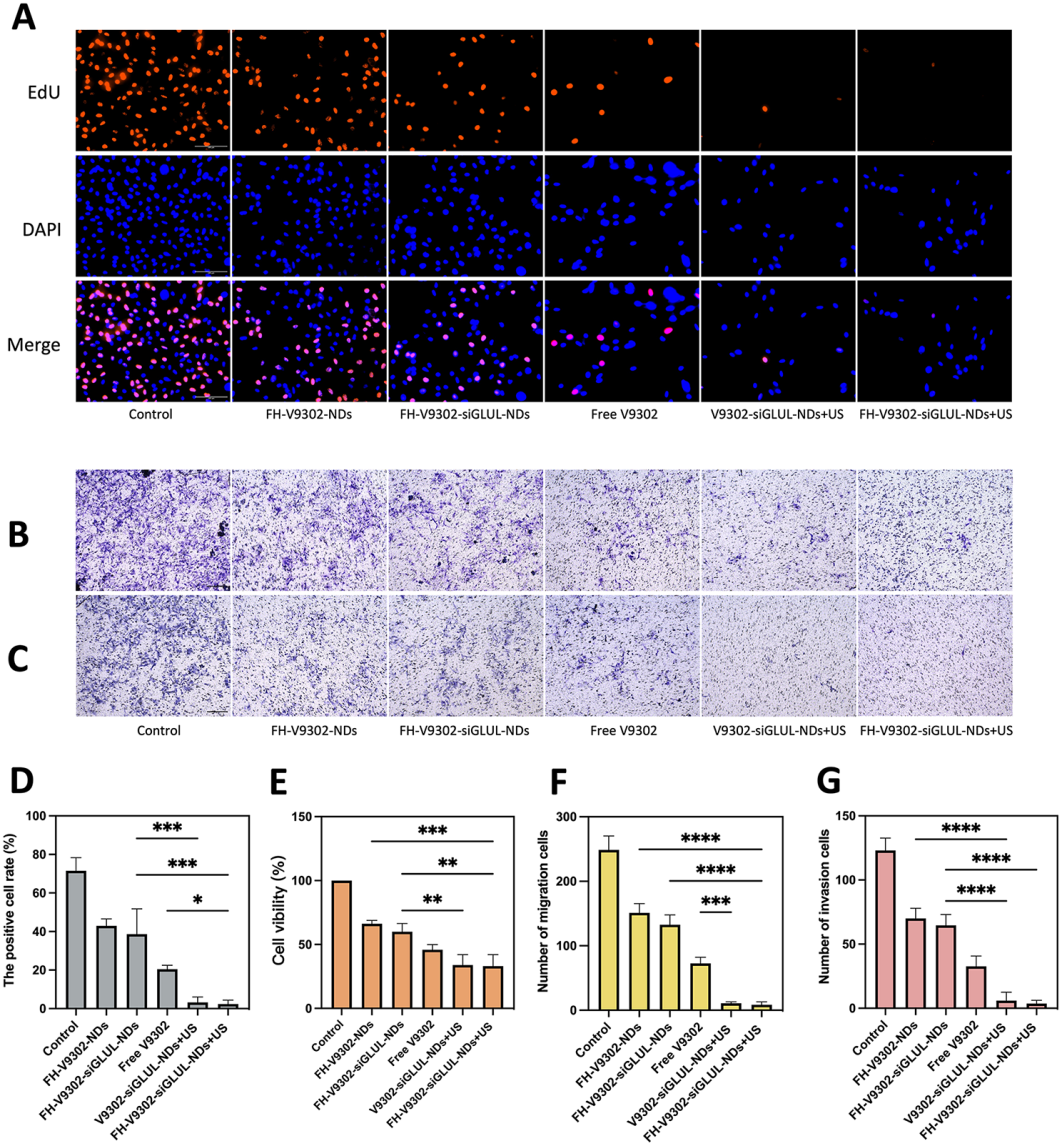
Anti-tumor ability in vitro. **(A)** Fluorescence images of EdU-positive cells after 24 h of various treatments. Scale bar: 100 μm. (B&C) Treated B16F10 cells were seeded on the upper chamber with or without matrigel. After 24 h invaded cells were detected and images captured and analyzed as described in Materials and Methods. Image of migration **(B)** and invasion **(C)** cells examined by Transwell assay. Scale bar: 200 μm. **(D)** Quantification of the percentage of EdU-positive cells. **(E)** Cell viability of B16F10 cells after 24 h of various treatments. (F&G) Quantification of the migrating cells **(F)** and invading cells**(G)**. *p < 0.05, **p < 0.01, ***p < 0.001, ****p < 0.0001(ANOVA test). All statistical data are expressed as means ± SD (n = 3)

### Antitumor efficacy in vitro

Blocking glutamine metabolism is a promising antitumor approach [37]. The IC50 values of free V9302 on B16F10 cells were 33.52 µM. At V9302 concentration of 35 µM, the efficacy of the combination treatment on the proliferation of B16F10 cells was confirmed by the EdU assay (Fig. 8A). As shown in Fig. 8D, the percentage of EdU-positive cells in the FH-V9302-siGLUL-NDs + US group was the lowest, at 2.35 ± 2.06%, and the V9302-siGLUL-NDs + US group was 3.18 ± 2.86%. Probably because the FH-short peptide did not have a significant targeting effect on B16F10 cells. The rate of EdU positivity was higher in the FH-V9302-siGLUL-NDs and FH-V9302-NDs groups than in the free V9302 group, probably because that V9302 was wrapped by nanodroplets and slowly released without ultrasound stimulation. The FH-V9302-siGLUL-NDs group inhibited proliferation to some extent than the FH-V9302-NDs group, probably due to the inhibition of GLUL [11]. Then, the cell viability was measured by the CCK8 method to measure the efficiency of different treatment (Fig. 8E). Compared with other groups, the cell viability of FH-V9302-siGLUL-NDs + US group was the lowest at 33.11 ± 9.01%, followed by V9302-siGLUL-NDs + US group at 33.94 ± 8.16% and FH-V9302-siGLUL-NDs at 59.86 ± 6.50%, indicating that the FH-short peptide had no effect on B16F10 and ultrasound stimulation promoted drug release greatly inhibiting cell viability. In conclusion, all groups containing V9302 obviously inhibited the proliferation and cell viability of B16F10 cells. All results suggested that UTMD combined with glutamine metabolic interruption had excellent anti-tumor capacity in vitro.

The migratory (Fig. 8B) and invasive (Fig. 8C) capacity of the cells was assessed in vitro using transwell assay. Figure 8 F showed the V9302-siGLUL-NDs + US group (11.00 ± 2.0) and the FH-V9302-siGLUL-NDs + US group (8.33 ± 4.50) had a low number of migrating cells. Comparatively, the FH-V9302-NDs, FH-V9302-siGLUL-NDs and free V9302 groups had 151.0 ± 14.42, 132.7 ± 15.18 and 73.67 ± 9.60 migrating cells, respectively. As shown in Fig. 8G, the results of the invasion assay showed similar trends to the migration assay. These findings suggested that UTMD combined with glutamine metabolism inhibition could effectively inhibit tumor migration and invasion ability. In conclusion, all groups containing V9302 could significantly inhibit the migration and invasion of B16F10. The presence or absence of FH-short peptide had little effect on the migration and invasion of B16F10 cells.

**Fig. 9:**
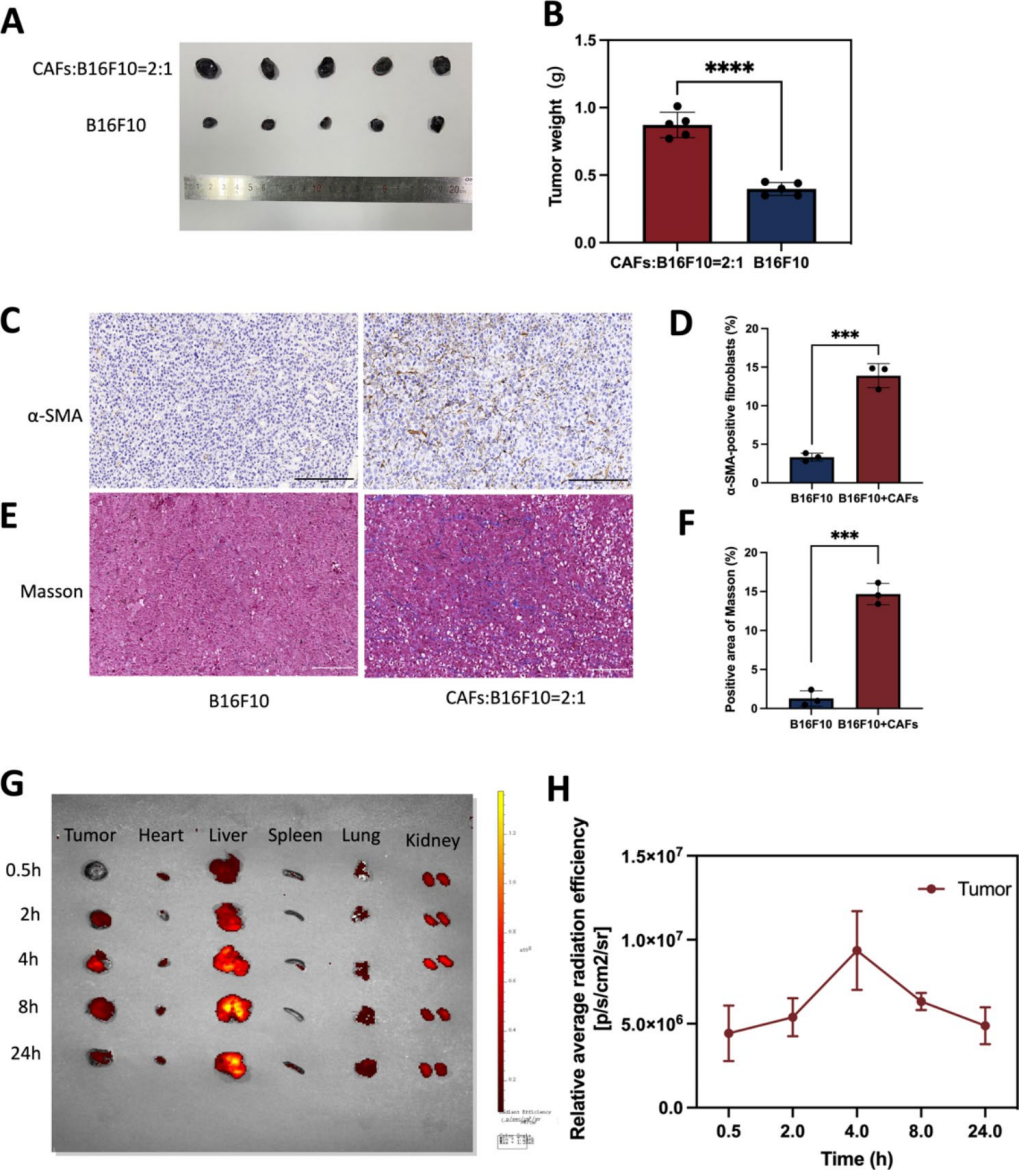
The establishment of a tumor model with co-implantation of B16F10 and CAFs cells and biodistribution assessment of nanodroplets. **(A)** B16F10 mixed with or without CAFs cells were injected intradermally into flanks of C57 mice, respectively. Photographs of tumor model after 10 days. **(B)** Statistics of tumor weight. **(C)** IHC staining of α-SMA. Scale bar: 200 μm. **(D)** Quantitative analysis of α-SMA using Image J software. **(E)** Masson trichrome staining. Scale bar: 200 μm. **(F)** Quantitative analysis of collagen deposition using Image J software. **(G)** Biodistribution of nanodrugs in the body 4 h after tail vein injection. **(H)** Quantitative analysis of biodistribution of nanodrugs in tumor. ***p < 0.0001, ****p < 0.0001(*t*-test). All statistical data are expressed as means ± SD (n = 3)

### Biodistribution of nanodrugs in vivo

In vitro, it has been shown that TGF-β1 can induce the conversion of NIH/3T3 into CAFs, then mimicking TME in vivo was the question to be investigated in this study. According to existing studies [38–40], B16F10 and CAFs cells were injected into mice at a 1:2 ratio in this experiment. Mouse models of co-inoculated melanoma was constructed with B16F10 mixed with or without CAFs cells, and 10 days later, tumors were collected and prepared for immunohistochemistry assess CAFs expression levels. The results showed that the tumor size of the mixed transplanted tumor group was significantly larger than that of the transplanted B16F10 alone (Fig. 9A). Statistics of tumor weight was showed in Fig. 9B(*p* < 0.0001). As shown in the Fig. 9D**&F**, co-cultured tumors contained approximately 13% CAFs in total control cells. Compared with the tumor model inoculated with B16F10 alone, α-SMA (a CAF-labeled protein) expression was increased in the tumor model inoculated with B16F10 mixed with CAFs (Fig. 9C). The expression of collagen secreted by CAFs was increased in tumor models inoculated with B16F10 mixed with CAFs compared to tumor models inoculated with B16F10 alone (Fig. 9E). The final co-culture model had a small proportion of CAFs and the majority of tumor cells, probably because the growth rate of cancer cells and CAFs cells was different. Tumor models co-cultured with B16F10 cells and CAFs cells is similar to the real melanoma in vivo more than tumor models with B16F10 alone. We also verified the syngeneic model by immunostaining for S100 expression. S100 calcium-binding protein B (S100-β) is a specific protein in melanoma. **Fig. S2** shows obvious positive rate in melanoma tumor. As this study used mouse melanoma and CAFs, future studies could delve into the results observed in TME of human melanoma.

Encouraged by the excellent tumor targeting ability of FH-V9302-siGLUL-NDs, the biodistribution of nanodroplets in a mouse tumor model was further investigated. The highest peak distribution of Dil-labelled FH-V9302-siGLUL-NDs was reached in the tumor 4 h after intravenous injection. Even after 24 h, fluorescent signals were still present in tumor tissues, indicating the accumulation of long-term retention of FH-V9302-siGLUL-NDs and deep penetration into the tumor (Fig. 9G). The fluorescence signal was quantified (Fig. 9H). Therefore, in subsequent experiments, US irradiation was performed on tumors 4 h after FH-V9302-siGLUL-NDs administration. In addition, the liver showed the highest fluorescence intensity among normal organs and accumulated gradually over time, indicating that the liver captured the lipid nanodroplets. We guessed that the interaction between positive nanodroplets and negative cell membranes effect the clearance rate [41, 42]. Although some studies have reported longer half-life for cationic MBs [43], it is important to balance their interaction and retention.

**Fig. 10:**
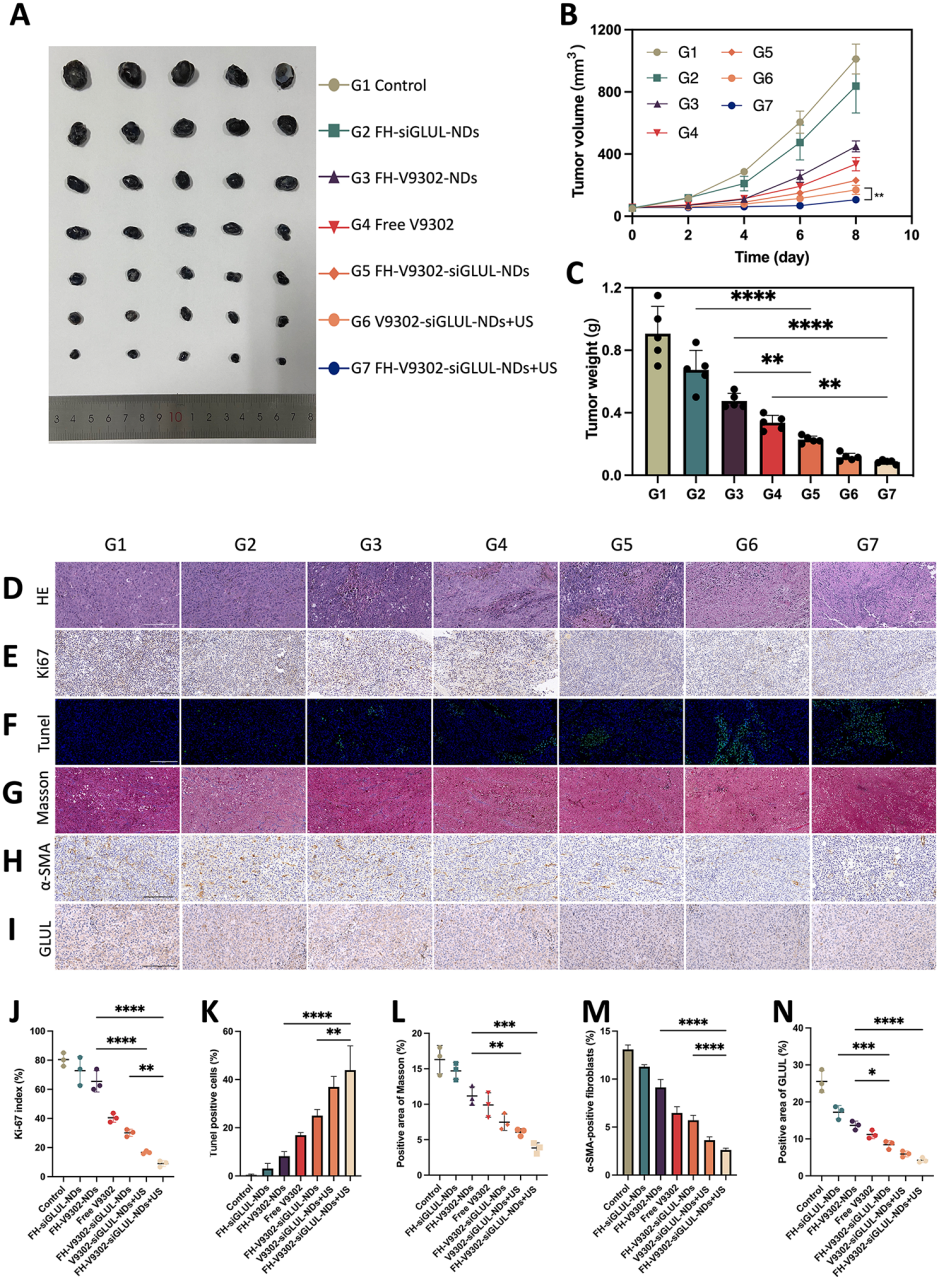
In vivo antitumor efficacy and reprogramming of TME. **(A)** Photographs of tumor formed by B16F10 and CAFs after four treatments. **(B)** Changes of tumor volume during the treatment (n = 5) **p < 0.001(*t*-test). **(C)** Weight of tumors formed by B16F10 and CAFs after four treatments (*n* = 5). **(D)** H&E staining. Scale bar: 200 μm. **(E)** Ki67 staining. Scale bar: 200 μm. **(F)** Tunel staining. Scale bar: 200 μm. **(G)** Masson trichrome staining. Scale bar: 200 μm. **(H)** IHC staining of α-SMA. Scale bar: 200 μm. **(I)** IHC staining of GLUL. Scale bar: 200 μm. **(J)** Ki67 index analysis (n = 3). **(K)** Quantitative analysis of Tunel positive cells (n = 3). **(L)** Analysis of positive area of Masson (n = 3). **(M)** Quantitative analysis of α-SMA (n = 3). **(N)** Analysis of positive area of GLUL (n = 3). * *p* < 0.05, ** *p* < 0.01, and *** *p* < 0.001 (ANOVA test). All statistical data are expressed as means ± SD (n = 5). G1, Control; G2, FH-siGLUL-NDs; G3, FH-V9302-NDs; G4, Free V9302; G5, FH-V9302-siGLUL-NDs; G6, V9302-siGLUL-NDs + US; and G7, FH-V9302-siGLUL-NDs + US

### In vivo antitumor efficacy

Female C57 mice carrying tumors were treated differently to investigate the anti-tumor effect. As shown in Fig. 10A and B, compared to the control, the FH-siGLUL-NDs group exhibited modest tumor inhibition. The all treated groups containing V9302, compared to the FH-siGLUL-NDs group, exhibited significant tumor suppression. We also found that the free V9302 group exhibited stronger tumor suppression than the FH-V9302-NDs group, which was mainly due to the slow-release ability of the nanodroplets without US stimulation. As expected, the FH-V9302-siGLUL-NDs + US-treated group showed the strongest tumor suppression, followed by V9302-siGLUL-NDs + US with 9.34% and 12.78% of tumor weight of the control group, respectively (Fig. 10C). Ultrasound combined with metabolic inhibition significantly inhibited tumor growth. Meanwhile, selective abrogation of glutamine metabolism in CAFs in vivo has been shown to inhibit the growth of ovarian tumors in mice [13]. In our experiments, significant inhibition of tumor growth was observed with V9302 treatment alone. Notably, the combination of V9302 plus siGLUL or V9302 plus siGLUL plus ultrasound stimulation resulted in greater inhibition of tumor growth, implying an additional effect of metabolic starvation treatment combined with blockade of glutamine synthesis from CAFs. Our work providing an opportunity to target both the CAFs and cancer cells to improve treatment outcomes.

To verify the anti-tumour effect, H&E (Fig. 10D), Ki-67(Fig. 10E) and Tunel (Fig. 10F) staining of tumour tissues was performed. Figure 10D showed FH-V9302-siGLUL-NDs + US group had the largest quantity of tumour cell necrosis of all groups. Ki-67 also showed it had the most pronounced inhibitory effect on tumor cell proliferation (Fig. 10J) and Tunel staining showed it had strongest pro-apoptotic effect (Fig. 10K). The above results suggested that combined ultrasound with remodeling of glutamine metabolism could exhibit good antitumor effects.

### TME remodeling

In order to reveal the mechanisms of the anti-tumour effects of TME glutamine metabolic reprogramming, TME was evaluated. Tumor ECM expression was assessed by immunohistochemistry (IHC) of α-SMA and Masson trichrome staining for collagen [44]. As shown in Fig. 10G and H, in control group, a significant amount of collagen and α-SMA positive fibroblasts was found settling in the intercellular stroma, while it was almost absent in the FH-V9302-siGLUL-NDs + US group. In addition, GLUL in tumors was also measured by IHC (Fig. 10I). All nanodroplets containing V9302 can be used in significantly reducing ECM expression. As shown in Fig. 10L, V9302-siGLUL-NDs + US and FH-V9302-siGLUL-NDs + US downregulated expression of collagen to 6.06 ± 0.50% and 3.83 ± 0.74%, respectively. As shown in Fig. 10M, V9302-siGLUL-NDs + US and FH-V9302-siGLUL-NDs + US downregulated expression of α-SMA to 3.64 ± 0.35% and 2.61 ± 0.19%, respectively. The stronger effect may be due to the fact that ultrasound stimulated the prompt release of V9302 and siGLUL and effectively eliminated ECM, thereby improving the nanodroplets infiltration in the tumor. As shown in Fig. 10N, V9302-siGLUL-NDs + US and FH-V9302-siGLUL-NDs + US downregulated expression of GLUL to 5.93 ± 0.80% and 4.23 ± 0.66%, respectively. The stronger effect of FH-V9302-siGLUL-NDs + US may be due to its better targeting to tumor stroma. Therefore, we concluded that metabolic inhibition of FH-V9302-siGLUL-NDs combined with UTMD could down-regulate GLUL and effectively degrade ECM.

## Conclusion

In this study, we successfully constructed a CAFs targeted drug delivery nanosystem co-loaded with ASCT2 (SLC1A5) inhibitor V9302 and GLUL siRNA. The designed FH-V9302-siGLUL-NDs + US treatment exhibited significant anti-tumor effects against melanoma with good tumor targeting, biosecurity and CEUS imaging properties. All these results were obtained by double suppression of ASCT2 (SLC1A5) and GLUL in B16F10 and CAFs cells with reprogramming of TME metabolism, which further eliminated tumor ECM and increased nanodrug penetration. The novel nanodroplets presented a good example to improve the metabolic treatment of various solid tumors. Additionly, further research needed to be explored, such as studies in different cell lines, metabolic aspects and immunological aspects. And we were currently unable to confirm the mechanisms of impact.

## Electronic supplementary material

Below is the link to the electronic supplementary material.

Supplementary Material 1

## Data Availability

The data of this study is available from the corresponding authors on reasonable request.
