# Supplementary material for: CAFs targeted ultrasound-responsive nanodroplets loaded V9302 and GLULsiRNA to inhibit melanoma growth via glutamine metabolic reprogramming and tumor microenvironment remodeling
Source: J Nanobiotechnology. 2023 Jul 8;21:214. doi: 10.1186/s12951-023-01979-z (PMC10329298; doi:10.1186/s12951-023-01979-z)
Supplement: Supplementary file 1 — Supplementary Material 1 [file 12951_2023_1979_MOESM1_ESM.docx]

**Supporting Information**

**CAFs targeted ultrasound-responsive nanodroplets loaded V9302 and GLULsiRNA to inhibit melanoma growth via glutamine metabolic reprogramming and tumor microenvironment remodeling**

Chen Ai, Xiao Sun, Shan Xiao, Lu Guo, Mengmeng Shang, Dandan Shi, Dong Meng, Yading Zhao, Xiaoxuan Wang, Jie Li^*^

*Corresponding authors: Tel/fax: +86-531-82166101. E-mail address: [jieli@email.sdu.edu.cn](mailto:jieli@email.sdu.edu.cn)

Department of Ultrasound, Qilu Hospital of Shandong University, Jinan, Shandong 250012, China


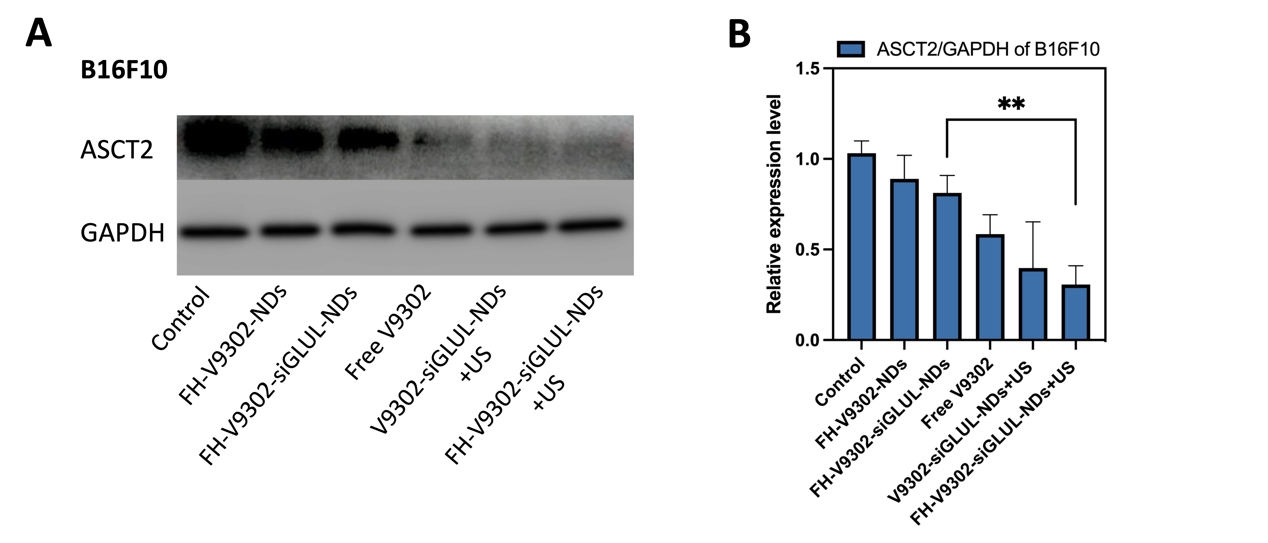


**Fig. S1.** (A) Western blot analysis of ASCT2 after 24 h of B16F10 treated with different elements. (B) Quantitative analysis of ASCT2 in B16F10 by image J (n=3). Results were shown as mean ± SD. ***p* < 0.01 (ANOVA test).


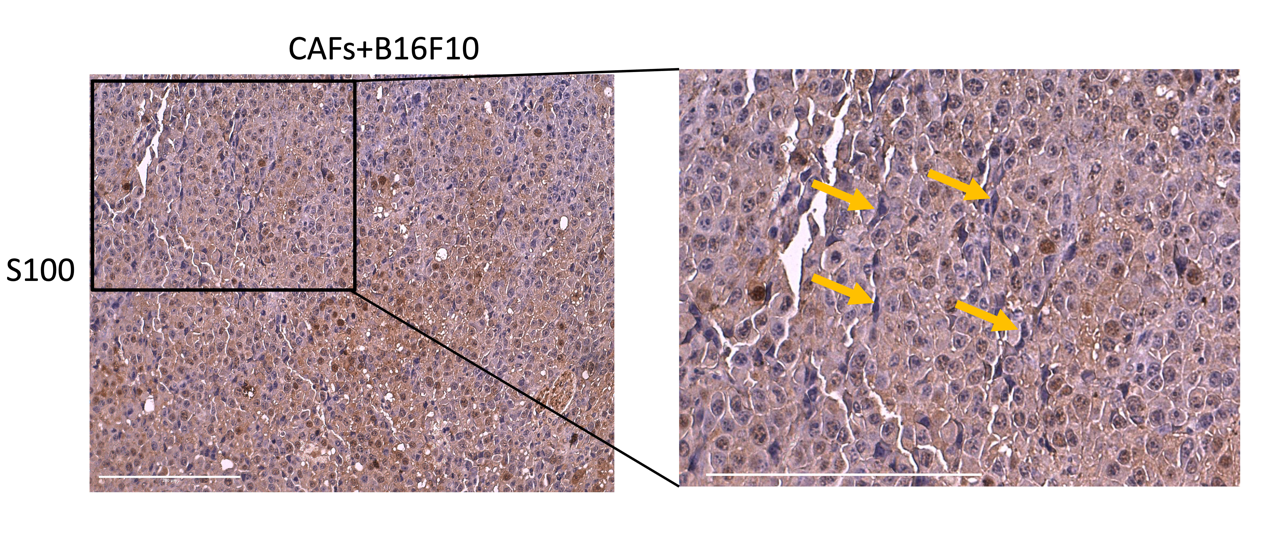


**Fig. S2.** Melanoma tumor by immunostaining for S100 expression. Scale bars: 200μm.Yellow arrow indicates CAFs.
